# Running after your mental health

**DOI:** 10.1016/j.eclinm.2025.103276

**Published:** 2025-05-21

**Authors:** eClinicalMedicine

Marathon runners, trail runners, avid Park Run-ners, or just late runners for the bus. Whatever the cause, running means something different to us all. 2025 marked record participation at the London marathon, with over 56,640 finishers, global levels of enthusiasm for marathons continue to grow. The majority of marathons are open to all, with Kathrine Switzer, in 1967, shattering the notion that only men should run, and the Oita International Wheelchair Marathon of 1981 paving the way for wheelchair participation to become standard in major events.

Although long-distance running comes with the elevated risk of dehydration, heat stroke, or fractures, people take part in marathons, and events such as Cancer Research UK's Race for life for various reasons. Perhaps in support of charity, for loved ones, to find community, and believe it or not, for the fun of experiencing the rush of endorphins and endocannabinoids and the elusive runners' high. In addition to a sense of achievement, running is a relatively inexpensive sport that offers physical benefits for numerous health conditions. While also promoting cardiovascular and respiratory health as well as bone strength, further benefits include reduced risk of cancer, type 2 diabetes, stroke, and a growing number of non-communicable diseases in those who exercise regularly.

In addition to physical health, we know that running, and physical activity in general, has strong benefits for our mental wellbeing. With a plethora of disorders negatively affecting an individual's mental health, depression and anxiety are among the most common. A recent analysis of 161,023 participants from The UK Biobank associates not only running, but low-intensity exercise such as walking, with a lower risk of depression and anxiety. It is not the distance or speed, but simply building a healthy relationship with exercise, that is important.

The WHO currently recommends 150 min of exercise per week. That is a brisk walk, jog or a run, less than 22 m per day. Anything that raises your heart rate, makes you breathe faster, and feel warmer counts. Despite the known benefits, over 1.8 billion adults are thought to be inactive, with additional global surveys of 1.6 million children, suggesting a disproportionately negative effect of such inactivity for young girls.

There are many physical and societal barriers that prevent regular exercise, including growing air pollution, social stigma, lifestyle, motivation, and accessibility. The WHO's Global Action Plan on Physical Activity 2018–30 (GAPPA) aims to promote worldwide campaigns to increase participation in physical activity at all levels. National policies such as the Australian Sport Commission and UK's All Our Health Policy continue to promote equity, diversity, and inclusion with dedicated areas for mental health in sport. Initiatives such as Sport England's This Girl Can campaign also continue to champion engagement of girls with exercise.

These initiatives are backed by increasing evidence associating running and other physical activities with improvement to overall wellbeing and reduced risk of depression and anxiety. Evidence can be found in recent studies, including a 2023 systematic review, correlating regular physical activity with a reduced risk of depression and anxiety. Further research, including a US-based cross sectional study of 1.2 million individuals and 60,000 UK-based Park Run participants, also showcase significant associations between positive mental health status and physical activity. Greater effects have also been reported in those from disadvantaged backgrounds and those with pre-existing health risks.

The MOTAR study of 141 individuals from the Netherlands has even suggested that, in some cases, running can have similar effects to antidepressants on overall wellbeing. Similar benefits were also seen amongst university students in both Austria as well as the UK's MINDFIT programme (based on the popular Couch to 5k initiative), where improvements to wellbeing were seen as early as 2 weeks.

Further evidence in adolescents associates changes in lifestyle, such as an increase in physical activity, with reduction of weight and reduced symptoms of anxiety and depression. Thus, highlighting the importance of a healthy relationship with diet and exercise from a young age. This is particularly important for young girls, as seen in global trends where on average young girls are thought to have access to lower levels of physical exercise than boys. A recent review in *eClinicalMedicine* has also suggested benefits of exercise, such as running and cycling, for the management of ADHD and associated mental health disorders in adolescents.

Not only does physical activity improve mental wellbeing, but increasing studies emphasize the social benefits of running through group settings and events to support improved mental health, motivation, and a sense of belonging. Research highlighting the benefits of exercising with friendswas recently documented in a BBC ideas film. Furthermore the Royal College of Physicians was recently praised for supporting the prescription of Park Run in the UK to support patients with physical and mental health disorders such as anxiety and depression. With similar physical activity prescription models being adopted throughout Europe.

While many of us might not be aiming to endure 26.2 miles anytime soon, through watching all of these incredible humans endure the physical and mental exertion of a marathon, often for causes bigger than themselves, perhaps we can be inspired to lace up our running shoes, grab our headphones, and head out for a run. Maybe this year, together, we can all run a little more in support of both our physical and mental wellbeing.

